# Venous thromboembolism risk in the postoperative interval during the COVID-19 pandemic: meta-analysis

**DOI:** 10.1093/bjsopen/zraf039

**Published:** 2025-04-15

**Authors:** Andrew Jackson, Christopher A Lewis-Lloyd, Oluwademilade Merotohun, Colin J Crooks, David J Humes

**Affiliations:** Nottingham Digestive Diseases Centre, Division of Translation Medical Sciences, School of Medicine, University of Nottingham, Queen’s Medical Centre, Nottingham, UK; National Institute for Health Research (NIHR) Nottingham Biomedical Research Centre, Nottingham University Hospitals NHS Trust and University of Nottingham, Queen’s Medical Centre, Nottingham, UK; Nottingham Digestive Diseases Centre, Division of Translation Medical Sciences, School of Medicine, University of Nottingham, Queen’s Medical Centre, Nottingham, UK; National Institute for Health Research (NIHR) Nottingham Biomedical Research Centre, Nottingham University Hospitals NHS Trust and University of Nottingham, Queen’s Medical Centre, Nottingham, UK; Nottingham Digestive Diseases Centre, Division of Translation Medical Sciences, School of Medicine, University of Nottingham, Queen’s Medical Centre, Nottingham, UK; National Institute for Health Research (NIHR) Nottingham Biomedical Research Centre, Nottingham University Hospitals NHS Trust and University of Nottingham, Queen’s Medical Centre, Nottingham, UK; Nottingham Digestive Diseases Centre, Division of Translation Medical Sciences, School of Medicine, University of Nottingham, Queen’s Medical Centre, Nottingham, UK; National Institute for Health Research (NIHR) Nottingham Biomedical Research Centre, Nottingham University Hospitals NHS Trust and University of Nottingham, Queen’s Medical Centre, Nottingham, UK; Nottingham Digestive Diseases Centre, Division of Translation Medical Sciences, School of Medicine, University of Nottingham, Queen’s Medical Centre, Nottingham, UK; National Institute for Health Research (NIHR) Nottingham Biomedical Research Centre, Nottingham University Hospitals NHS Trust and University of Nottingham, Queen’s Medical Centre, Nottingham, UK

## Abstract

**Background:**

During the COVID-19 pandemic, global trends emerged, indicating increased venous thromboembolism (VTE) incidence among postoperative patients, potentially attributable to perioperative COVID-19 infection. However, there are insufficient data on VTE incidence among postoperative patients in the context of the pandemic. The aim of this study was to examine the global incidence of postoperative VTE during the COVID-19 pandemic.

**Methods:**

A systematic search of MEDLINE and Embase databases, as well as three other registered databases, was conducted from 1 January 2019 to 3 November 2023, with pre-registration in PROSPERO, the international prospective register of systematic reviews (CRD42023460464). Any study reporting patients aged ≥18 years undergoing surgery during the COVID-19 pandemic was included. Outcomes were aggregated absolute and unadjusted relative risks, plus incidence rates per 1000 person-years, of 30- or 90-day postoperative VTE in patients operated on before or during the COVID-19 pandemic and those with or without perioperative COVID-19 infection during the pandemic.

**Results:**

Of 5943 studies, 17 were available for meta-analysis, reporting on 3 035 037 patients. VTE incidence rates in perioperative COVID-19-positive compared with COVID-19-negative patients were significantly higher after total joint arthroplasty (244 (95% c.i. 110 to 541) *versus* 71 (95% c.i. 47 to 108) per 1000 person-years), other orthopaedic surgery (253 (95% c.i. 240 to 266) *versus* 138 (95% c.i. 84 to 229) per 1000 person-years), and emergency general and gastrointestinal surgery (474 (95% c.i. 226 to 995) *versus* 97 (95% c.i. 61 to 157) per 1000 person-years). No significant differences in VTE rates were reported in studies comparing pre-pandemic and pandemic VTE incidence rates.

**Conclusion:**

There were consistent increased VTE rates in perioperative COVID-19-positive patients, particularly those undergoing orthopaedic surgery, and emergency general and gastrointestinal surgery. Further investigation is required to delineate postoperative VTE risk and how it varies by COVID-19 variant and vaccination to inform future practice.

## Teaser

This systematic review and meta-analysis investigates the incidence of postoperative venous thromboembolism (VTE) during the COVID-19 pandemic, revealing significantly higher rates in patients with perioperative COVID-19 compared with those without. Data from 17 studies involving >3 million patients showed VTE rates were raised in those undergoing total joint arthroplasty, other orthopaedic surgery, and emergency general and gastrointestinal surgery. The findings underscore the need for further research to understand VTE risk variations related to COVID-19 variants and vaccination.

## Introduction

Annually, it is estimated that >300 million surgical procedures are performed globally^1^. Venous thromboembolism (VTE), which includes deep-vein thrombosis (DVT) and pulmonary embolism (PE), is a recognized and potentially preventable postoperative complication^2^. Although these events are relatively rare, they can lead to significant patient morbidity and, in some cases, mortality, along with an economic burden on healthcare systems^3^.

After the emergence of the COVID-19 pandemic, it became evident that patients with the infection were at increased risk of VTE, despite receiving prescribed prophylaxis^4^. Incidence rates were reported to range from 9% to 26%^5,6^, with higher frequencies observed in patients admitted to ICUs^7^. The coagulopathy observed in COVID-19-positive patients is multifaceted and attributed to the complex interplay of inflammation, endothelial dysfunction, a milder form of disseminated intravascular coagulation, and microvascular thrombosis^8^.

The COVIDSurg study on VTE reported on the incidence of VTE within 30 days after both elective and emergency surgery for any indication. The study’s findings indicated a significantly increased risk of postoperative VTE in patients with perioperative COVID-19 infection, with an adjusted OR of 1.48 (95% c.i. 1.08 to 2.03), and in those with a recent infection, with an adjusted OR of 1.96 (95% c.i. 1.16 to 3.33)^9^. However, this study occurred early in the pandemic (October 2020) and had a high proportion of emergency patients, approximately one third (30%), and so may not be fully representative of the true VTE risk in this group.

To date, no systematic review or meta-analysis exists that examines the differences in risk of postoperative VTE during the COVID-19 pandemic and of those with perioperative COVID-19 infection. The aim of this study was to thoroughly examine and quantify the incidence of postoperative VTE among patients undergoing surgical procedures worldwide during the COVID-19 pandemic.

## Methods

The review protocol was prospectively registered before starting the systematic review in PROSPERO, the international prospective register of systematic reviews (CRD42023460464; https://www.crd.york.ac.uk/PROSPERO/view/CRD42023460464), and was conducted in accordance with PRISMA and MOOSE (‘Meta-analysis Of Observational Studies in Epidemiology’) guidelines^10,11^ (see *Fig. S1* for the PRISMA checklist and *Fig. S2* for the MOOSE checklist). No other review protocol was registered for this study.

### Data sources and searches

A systematic search was conducted in MEDLINE and Embase databases using the Ovid platform, as well as the Cochrane Library, US National Library of Medicine ClinicalTrials.gov, and International Clinical Trials Registry Platform databases. The search targeted articles published from 1 January 2019 to 3 November 2023. Grey literature was explored through Google Scholar, with a comprehensive hand search of reference lists of included papers and recent systematic reviews. The search strategies were limited to English language studies, with no geographical restrictions applied (see *Fig. S3* for the full database search strategies).

### Eligibility criteria

#### Inclusion criteria

Studies focusing on adult patients (aged ≥18 years) undergoing surgical procedures in an operating environment during the COVID-19 pandemic were included. Prospective studies recruiting patients from 1 January 2019 to 3 November 2023 were considered to capture early data potentially relevant at the onset of the pandemic. Eligible studies needed to report a minimum of ten patients per cohort grouping.

#### Exclusion criteria

Studies were excluded if they included patients <18 years of age, those not undergoing surgical procedures during the study interval, or cases limited to endoscopic procedures. Studies failing to report postoperative VTE incidence (DVT and PE), case reports, and letters were also excluded.

### Study selection and data extraction and management

Two independent reviewers (A.J. and O.M.), blinded to each other’s assessments, used the Rayyan™ online interface to screen titles and abstracts, with discrepancies resolved through a third reviewer (C.A.L.-L.).

Data from included studies were extracted using a standardized pro forma. Extracted data encompassed authorship, year of publication, study design, country, database used, postoperative follow-up duration, study interval, surgical specialty, and types of surgery performed. Additionally, data on total patient numbers and perioperative COVID-19 status, as well as information on VTE, including definitions used, total event counts before and during the pandemic, overall incidence rates, and temporal association of VTE with COVID-19 positivity, were extracted.

To accurately ascertain the absolute rates of VTE and duration of associated risk, the authors focused on selecting studies with representative population-based samples that provided both the number of VTE events and the duration of follow-up. The study designs included in the analysis encompassed randomized clinical trials (RCTs) and large population-based database or registry cohort studies, with both regional and national scopes. Additionally, multicentre cohort studies were included, with the stipulation that the minimum number of patients per stratified grouping was ten or greater.

In preparation for the analysis, selected studies were systematically stratified based on several key parameters. These included the epidemiological design of each study, the duration of postoperative follow-up, and surgical specialties, along with specific procedures when available. A crucial element of the authors’ stratification process was identifying studies that conducted comparative analyses between pre-pandemic and pandemic intervals. Furthermore, the stratification considered the urgency of the surgical interventions, categorizing the patient cohorts into either elective or emergency cases.

When studies did not specify an absolute number of VTE events, the combined incidence of PE and DVT outcomes was used.

### Risk of bias

To assess the risk of bias, a modified Newcastle–Ottawa scale was used for population-based cohort studies and the Cochrane risk-of-bias tool was used for RCTs (see *Table S1* and *Fig. S4* for the risk-of-bias assessment).

### Statistical analysis

Studies available for analysis were initially divided according to specialty-specific surgery into the following groups: lower limb total arthroplasty, other orthopaedic surgery, emergency general and gastrointestinal surgery (EGS), and multispecialty surgery. Studies were further divided into those reporting comparisons between patient cohorts operated on during the COVID-19 pandemic with or without perioperative COVID-19 infection and/or those operated on before the COVID-19 pandemic (pre-pandemic) and those operated on during the pandemic.

The primary outcomes were the aggregated numbers and absolute incidence risks presented as percentages, with the related unadjusted relative risks of combined 30- and 90-day postoperative VTE derived from the absolute risks, as well as the pooled incidence rates of VTE at 30- and 90-days post-surgery, expressed per 1 000 person-years, accompanied by their respective 95% confidence intervals. For the meta-analysis, the VTE incidence rates from each study were transformed on a logarithmic scale to ensure uniformity and the standard error was calculated (1/√ (VTE events)). The DerSimonian and Laird random-effects model was employed due to its capability to accommodate the anticipated variability among studies, which were likely to differ in terms of populations, methodologies, and contexts. For data aggregation, the inverse-variance method was utilized to estimate heterogeneity, prioritizing studies with more precise data by inversely weighting each study’s contribution based on the square of its standard error (1/(standard error)^2^). This approach ensured a balanced representation across studies with diverse sample sizes and event rates and has previously been used^12^. The extent of heterogeneity among the included studies was quantitatively assessed using the *I*^2^ statistic. Data analysis and figure formation was performed using STATA^®^ SE version 18.5 (StataCorp, College Station, Texas, USA).

## Results

### Summary of included studies

**Fig. 1:**
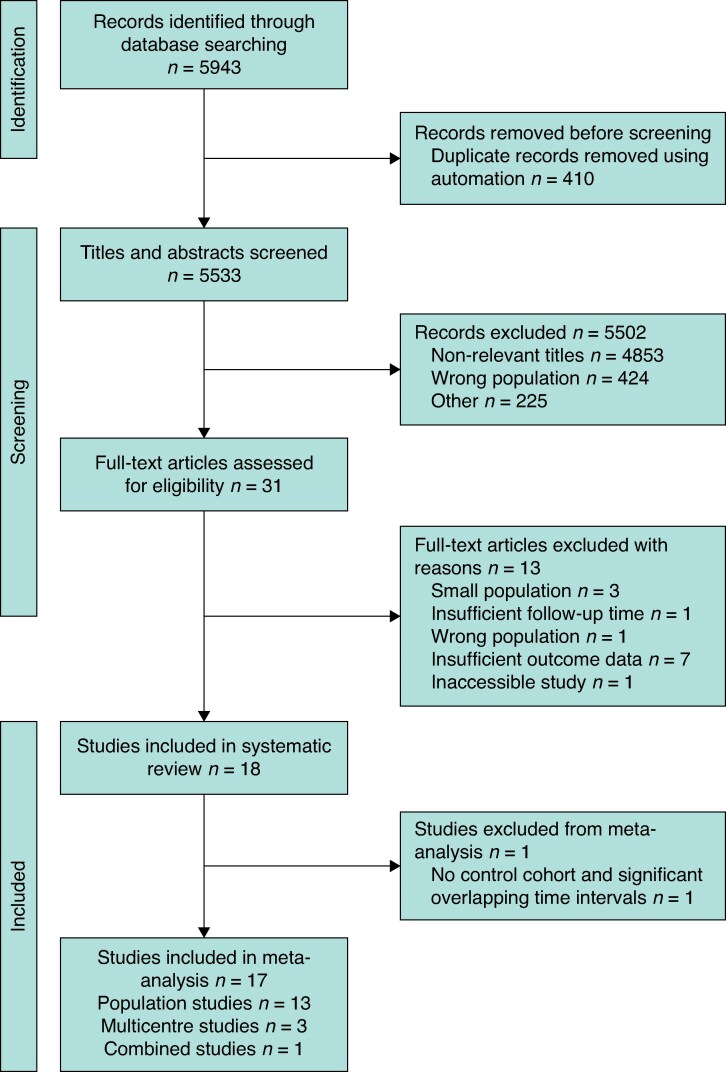
PRISMA flow diagram

In this systematic review across five electronic databases, 5943 studies were found, with 5533 titles and abstracts screened and 31 full-text publications appraised; 17 were included in the quantitative analysis and stratified for meta-analysis, as illustrated in *Fig. 1* (the PRISMA flow diagram), comprising 13 large database cohorts^13–25^, 3 multicentre cohorts^9,26,27^, and 1 combined population and multicentre study^28^, reporting on 3 035 037 patients (2 899 716, 133 635, and 1686 patients respectively). *Table 1* details the characteristics of the studies within the meta-analysis that are grouped according to the surgical procedures undertaken (see *Table S2* for a description of the studies included in the qualitative analysis).

**Table 1:** Description of the studies included in the meta-analysis

Study	Data source (country)	Follow-up (days)	Study time frame	Surgical procedures (proportion of elective operations)	VTE type	Total population	Overall number of VTE events (VTE incidence risk, %)	Cohort definition	
								Interval	COVID-19 positivity
**Description of orthopaedic TKA and THA studies**									
Badin *et al*.^22^ (2022)	ACS-NSQIP (USA & Canada)	30	Jul–Dec 2019 & Jul–Dec 2020	THA & TKA (100% elective)	DVT & PE	100 180	787 (0.8)	Pre-pandemic: Jul–Dec 2019 Pandemic: Jul–Dec 2020	
Forlenza *et al*.^24^ (2022)	Mariner & PearlDiver (USA)	90	Jan 2018–Apr 2020	THA & TKA (not specified)	DVT & PE	5680	229 (4.0)		≤90 days postop ICD-10 U07.1 code
Heckmann *et al*.^17^ (2023)	Premier Healthcare Database (USA)	90	Jan–Dec 2020	THA & TKA (100% elective)	DVT & PE	4984	14 (0.3)		±90 days periop ICD-10 U07.1 code
Heo *et al*.^13^ (2024)	IBM MarketScan & Medicare (USA)	90	Jan 2018–Dec 2021	Revision THA & TKA (not specified)	DVT & PE	660	24 (3.6)		≤90 days postop ICD-10 U07.1 & J12.82 codes
Lee *et al*.^19^ (2023)	Mariner & PearlDiver (USA)	90	Jan 2020–Jan 2021	THA & TKA (not specified)	DVT & PE	7230	220 (3.0)		≤90 days preop ICD-10 U07.1 code
Okewunmi *et al*.^14^ (2024)	Medicare (USA)	90	Jan 2016–Sep 2021	THA & TKA (100% elective)	DVT, PE, & VTE	2 422 051	50 600 (2.1)	Pre-pandemic: Jan 2016–Jan 2020 Pandemic: Feb 2020–Sep 2021 (includes post-vaccine interval)	Prior history of COVID-19 ICD-10 U10, U08, U09, U07.1, U09.9, & Z86.16 codes
Villa *et al*.^25^ (2022)	Cleveland Clinic Florida (USA)	30	Dec 2018–Mar 2021	THA & TKA (not specified)	VTE	19 068	312 (1.6)	Pre-pandemic: Dec 2018–Dec 2019 Pandemic: Jan 2020–Mar 2021	
Wenzel *et al*.^15^ (2024)	ACS-NSQIP (USA & Canada)	30	Jan–Dec 2021	THA & TKA (100% elective)	DVT & PE	2340	36 (1.5)		≤30 days postop ICD-10 U07.1 code (U07.2 excluded)
**Description of other orthopaedic surgery studies**									
Johnson *et al*.^18^ (2023)	TriNetX Research Network (international)	90	Apr 2020–Jan 2022	Arthroscopic, total joint arthroplasty, lumbar fusion, upper extremity, foot, & ankle (not specified)	VTE	48 084	2486 (5.2)		7–90 days preop ICD-10 U07.1–2 & J12.82 codes
Mercier *et al*.^20^ (2023)	ACS-NSQIP (USA & Canada)	30	Jan–Dec 2021	Total joint arthroplasty, trauma surgery, spinal surgery, sports surgery, & shoulder surgery (88.28% elective (COVID-19: negative, 88.39%; and positive, 57.70%))	VTE	194 121	1710 (0.9)		≤14 days preop ICD-10 U07.1 code
Song *et al*.^21^ (2023)	ACS-NSQIP (USA & Canada)	30	2019–2020	Lumbar fusion (100% elective)	DVT & PE	27 446	314 (1.1)	Pre-pandemic: 2019 Pandemic: 2020	
**Description of EGS studies**									
Chen *et al*.^16^ (2023)	ACS-NSQIP (USA & Canada)	30	Apr 2019–Dec 2020	Colorectal, abdominoperineal resection, low anterior resection, total proctocolectomy, & ostomy creation/revision (86.2% elective (pre-pandemic, 87.3%; and pandemic, 84.8%))	VTE	62 393	1111 (1.8)	Pre-pandemic: Apr–Dec 2019 Pandemic: Apr–Dec 2020	
Osorio *et al*.^27^ (2021)	COVID-CIR multicentre cohort study (Spain)	30	Mar–Jun 2019 & Mar–Jun 2020	Perianal surgery, hernia/eventration repair, appendectomy, cholecystectomy, colectomy, intestinal resection, lysis of adhesions or internal hernia repair, gastrointestinal perforation suture, splenectomy, minor liver trauma, gastrectomy, biliary surgery, exploratory laparotomy, other surgery, damage control surgery, pancreatectomy, pancreatic necrosectomy, & emergency laparotomy (0% elective)	VTE	5307	48 (0.9)	Pre-pandemic: Mar–Jun 2019 Pandemic: Mar–Jun 2020	≤15 days preop to 30 days postop RT-PCR or clinical diagnosis with CT positivity entry
**Description of multispecialty surgery studies**									
Argandykov *et al*.^28^ (2023)	ACS-NSQIP 2012 cohort & COVIDSurg multicentre cohort study (USA only)	30	Feb–Jul 2020	Neurosurgery, general surgery, thoracic, plastics, gynaecology, ENT surgery, cardiac surgery, urology, orthopaedics, and vascular surgery (26.7% elective (COVID-19: negative, 25.3%; and positive, 28.2%))	DVT & PE	1686	74 (4.4)		≤7 days preop to 30 days postop RT-PCR, rapid antigen, immunoglobulin, CT, clinical diagnosis positive entry
COVIDSurg Collaborative and GlobalSurg Collaborative^9^ (2022)	COVIDSurg multicentre cohort study (international)	30	Oct–Nov 2020	Neuro, thoracic, colorectal, oncology, orthopaedic, spinal, cardiac, HPB, ENT, gynaecology, obstetric, vascular, general, hernia, oesophagogastric, plastic, other, dental, breast, endocrine surgery, & ophthalmology (70.0% elective (COVID-19: negative, 70.5%; and positive, 54.9%))	VTE	128 009	742 (0.6)		Periop: ≤7 days preop to 30 days postop Recent: 7–42 days preop Previous: >42 days preop RT-PCR, rapid antigen, immunoglobulin, CT, clinical diagnosis positive entry
Deng *et al*.^23^ (2022)	Symphony Health COVID-19 Research (USA)	30	May 2019–May 2021	AAA repair (open/endovascular), CABG, CEA, colonic surgery, oesophagogastric, liver resection, hip and knee arthroplasty, laminectomy, spinal arthrodesis, hysterectomy, lung resection, neurosurgery, pancreatic resection, & prostatectomy (100% elective)	DVT & PE	5479	135 (2.5)	Pre-pandemic: May 2019–Jan 2020 Pandemic: Mar 2020–May 2021 (all COVID-19 positive)	Periop: 0–4 weeks postop Early: 4–8 weeks postop Late: >8 weeks postop ICD-10 U07.1 code
Jonker *et al*.^26^ (2021)	National multicentre cohort study (Netherlands)	30	Feb–Jun 2020	Wall exploration, adnexal surgery, adrenalectomy, lower limb amputation, appendectomy, arthrotomy axillary lymph node dissection, brachiocephalic fistula, neck dissection, cholecystectomy, colectomy, escharotomy, melanoma excision, external fixation lower and upper extremity, gastrectomy, gastroschisis repair, gastrostomy, hysterectomy, ileocaecal resection, inguinal hernia repair, inguinal lymph node dissection, intramedullary nailing proximal femur, intramedullary nailing tibia, biliary surgery, kidney transplantation, knee ligament repair, laparoscopy, laparotomy, liver resection, liver transplantation, lobectomy, lymph node biopsy, mastectomy/lumpectomy, mediastinoscopy, nephrectomy, oesophageal resection, orchiectomy, ORIF–femoral neck, pancreatic resection, parathyroidectomy, perianal abscess/fistula, permanent dialysis catheter insertion, placenta removal, pyloromyotomy, radiological drainage, rectopexy, small bowel resection, resection soft tissue tumour, caesarean section, sigmoid resection, splenectomy, tracheotomy, trauma, vascular bypass, VATS empyema drainage, VATS pleurectomy, venous access port placement, & wedge resection (64.9% elective (COVID-19, negative, 66.8%; and positive, 61.8%))	DVT & PE	319	5 (1.6)		≤30 days preop to ≤30 days postop RT-PCR or clinical diagnosis with CT positivity entry

VTE, venous thromboembolism; TKA, total knee arthroplasty; THA, total hip arthroplasty; ACS-NSQIP, American College of Surgeons National Surgical Quality Improvement Programme; DVT, deep-vein thrombosis; PE, pulmonary embolism; RT-PCR, reverse transcription polymerase chain reaction; ENT, ear, nose, & throat; HPB, hepatopancreatobiliary; AAA, abdominal aortic aneurysm; CABG, coronary artery bypass graft; CEA, carotid endarterectomy; ORIF, open reduction internal fixation; VATS, video-assisted thoracoscopic surgery ICD-10 codes: J12.82; pneumonia due to COVID-19; U07.1, COVID-19, virus identified (confirmed by laboratory testing); U07.2, COVID-19, virus not identified (diagnosed clinically or epidemiologically); U08, personal history of COVID-19; U09, post-COVID-19 condition; U09.9, post-COVID-19 condition, unspecified; U10, multisystem inflammatory syndrome associated with COVID-19; and Z86.16; personal history of infectious and parasitic diseases (personal history of COVID-19).

### Description of included studies

Of the 17 studies included in the meta-analysis, 13 studies originated from the USA or the USA and Canada^13–17,19–25,28^, 2 studies were international, based in the UK^9^ and USA^18^ respectively, 1 study originated from Spain^27^, and 1 study originated from the Netherlands^26^. Of the studies, 11 studies focused on orthopaedic surgical populations, with 8 studies dedicated to total hip arthroplasty (THA) and total knee arthroplasty (TKA)^13–15,17,19,22,24,25^; the 3 remaining orthopaedic studies expanded the scope to include arthroscopy and surgery of the foot, ankle, shoulder, and spine^18,20,21^. Of the 17 studies, 2 studies investigated patients undergoing EGS^16,27^ and 4 studies included patients undergoing multispecialty surgery^9,23,26,28^. Of the 17 studies, 4 studies divided their populations into pre-pandemic and pandemic intervals^16,21,22,25^, 10 studies stratified their patients according to COVID-19 infection status^9,13,15,17–20,24,26,28^, and 3 studies did both^14,23,27^. The sample sizes of the included studies varied significantly, ranging from 319 to 2 422 051, covering populations from the years 2012 and 2016 to 2022. Postoperative follow-up ranged between 30 and 90 days.

### Meta-analysis

#### Frequency and pooled incidence of VTE events after orthopaedic surgery according to COVID-19 infection status

**Fig. 2:**
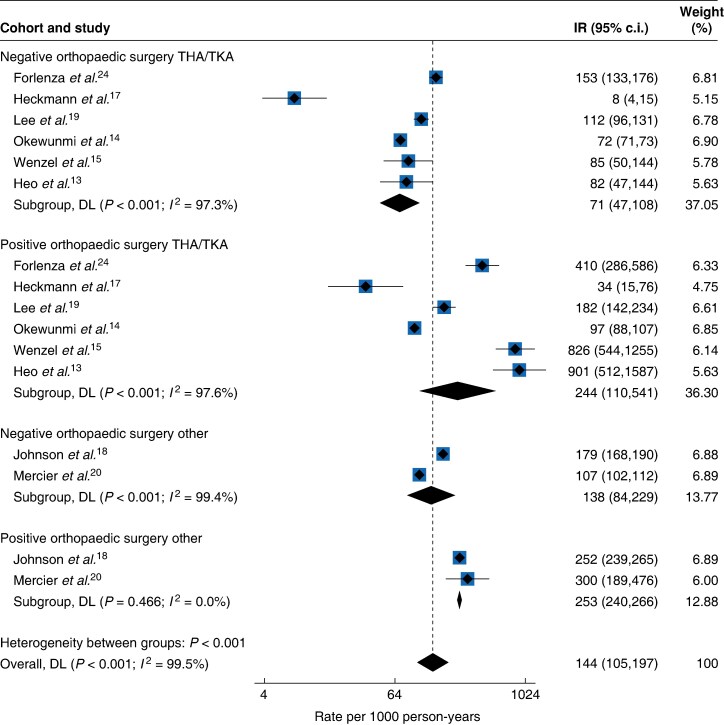
Postoperative VTE incidence rates per 1000 person-years after orthopaedic surgery according to COVID-19 infection status VTE, venous thromboembolism; IR, incidence rate; THA, total hip arthroplasty; TKA, total knee arthroplasty; DL, DerSimonian-Laird.

In the cohort of patients undergoing THA or TKA orthopaedic surgery, six studies^13–15,17,19,24^ examined the effect of COVID-19 infection on the rate of VTE (*Table 2*). Among these, COVID-19-positive patients demonstrated a 1.53-fold increase in aggregated unadjusted relative VTE risk compared with COVID-19-negative patients, with aggregated absolute risks of 2.7% (516 out of 19 066) *versus* 1.8% (11 145 out of 629 885) respectively. The overall pooled incidence rate was 244 (95% c.i. 110 to 541, *I*^2^ = 97.6%) per 1000 person-years in COVID-19-positive patients and 71 (95% c.i. 47 to 108, *I*^2^ = 97.3%) per 1000 person-years in the COVID-19-negative patient cohort (*Fig. 2*).

**Table 2:** Postoperative VTE incidence risks and rates of the included studies according to COVID-19 infection status and according to pre-pandemic and pandemic intervals

Study	Follow-up (days)	Pre-pandemic interval, VTE/population (incidence risk, %)	Pandemic interval, VTE/population (incidence risk, %)	COVID-19 infection status		VTE incidence rate per 1000 person-years (95% c.i.)			
				Negative, VTE/population (incidence risk, %)	Positive, VTE/population (incidence risk %)	Pre-pandemic	Pandemic	COVID-19 negative	COVID-19 positive
**Orthopaedic THA/TKA**									
Badin *et al*.^22^ (2022)	30	443/55 405 (0.8)	344/44 775 (0.8)			98 (89,107)	94 (84,104)		
Forlenza *et al*.^24^ (2022)	90			199/5368 (3.7)	30/312 (9.6)			153 (133,176)	410 (286,586)
Heckmann *et al*.^17^ (2023)	90			8/4272 (0.2)	6/712 (0.8)			8 (4,15)	34 (15,76)
Heo *et al*.^13^ (2024)	90			12/600 (2.0)	12/60 (20)			82 (47,144)	901 (512,1587)
Lee *et al*.^19^ (2023)	90			159/5840 (2.7)	61/1390 (4.4)			112 (96,131)	182 (142,234)
Okewunmi *et al*.^14^ (2024)	90	39 462/1 797 052 (2.2)	11 138/628 227 (1.8)	10 753/611 800 (1.8)	385/16 257 (2.4)	90 (89,91)	73 (71,74)	72 (71,73)	97 (88,107)
Villa *et al*.^25^ (2022)	30	119/9545 (1.3)	193/9523 (2.0)			153 (128,183)	249 (216,287)		
Wenzel *et al*.^15^ (2024)	30			14/2005 (0.7)	22/335 (6.6)			85 (50,144)	826 (544,1255)
**Orthopaedic other surgery**									
Johnson *et al*.^18^ (2023)	90			1037/24 042 (4.3)	1449/24 042 (6.0)			179 (168,190)	252 (239,265)
Mercier *et al*.^20^ (2023)	30			1692/193 381 (0.9)	18/740 (2.4)			107 (102,112)	300 (189,476)
Song *et al*.^21^ (2023)	30	151/14 973 (1.0)	163/12 473 (1.3)			123 (105,145)	160 (137,187)		
**EGS**									
Chen *et al*.^16^ (2023)	30	599/34 810 (1.7)	512/27 583 (1.9)			211 (195,229)	228 (209,249)		
Osorio *et al*.^27^ (2021)	30	24/2992 (0.8)	24/2315 (1.0)	17/2132 (0.8)	7/183 (3.8)	98 (66,146)	127 (85,189)	97 (61,157)	474 (226,995)
**Multispecialty surgery**									
Argandykov *et al*.^28^ (2023)	30			16/843 (1.9)	58/843 (6.9)			233 (143,381)	867 (670,1121)
COVIDSurg Collaborative and GlobalSurg Collaborative^9^ (2022)	30			666/123 591 (0.5)	76/4418 (1.7)			66 (61,71)	211 (169,264)
Deng *et al*.^23^ (2022)	30	53/2621 (2.0)	82/2858 (2.9)	53/2621 (2.0)	82/2858 (2.9)	266 (203,349)	379 (306,471)	266 (203,349)	379 (306,471)
Jonker *et al*.^26^ (2021)	30			0/196 (0.0)	5/123 (4.1)			0 (0,0)	505 (210,1213)

VTE, venous thromboembolism; THA, total hip arthroplasty; TKA, total knee arthroplasty; EGS, emergency general and gastrointestinal surgery.

Two studies^18,20^ included patients undergoing other (non-exclusively THA or TKA) orthopaedic procedures, including arthroscopy, total joint replacement, trauma surgery, and surgeries of the foot and ankle (*Table 2*). Within this procedural subgroup, aggregated analysis revealed an absolute VTE risk of 5.9% (1467 out of 24 782) among COVID-19-positive patients, compared with an aggregated absolute risk of 1.3% (2729 out of 217 423) among patients without the infection, an unadjusted 4.72-fold relative risk increase. The pooled incidence rates for this subgroup were 253 (95% c.i. 240 to 266, *I*^2^ = 0.0%) per 1000 person-years among COVID-19-positive patients and 138 (95% c.i. 84 to 229, *I*^2^ = 99.4%) per 1000 person-years among the COVID-19-negative group (*Fig. 2*).

#### Frequency and pooled incidence of VTE events after orthopaedic surgery comparing pre- pandemic and pandemic intervals

Within the orthopaedic surgery cohort, four studies^14,21,22,25^ stratified their populations into pre-pandemic and pandemic intervals (*Table 2*). There were three studies that included patients undergoing THA and TKA procedures, and a single study exclusively examined spinal (lumbar) surgery^21^.

**Fig. 3:**
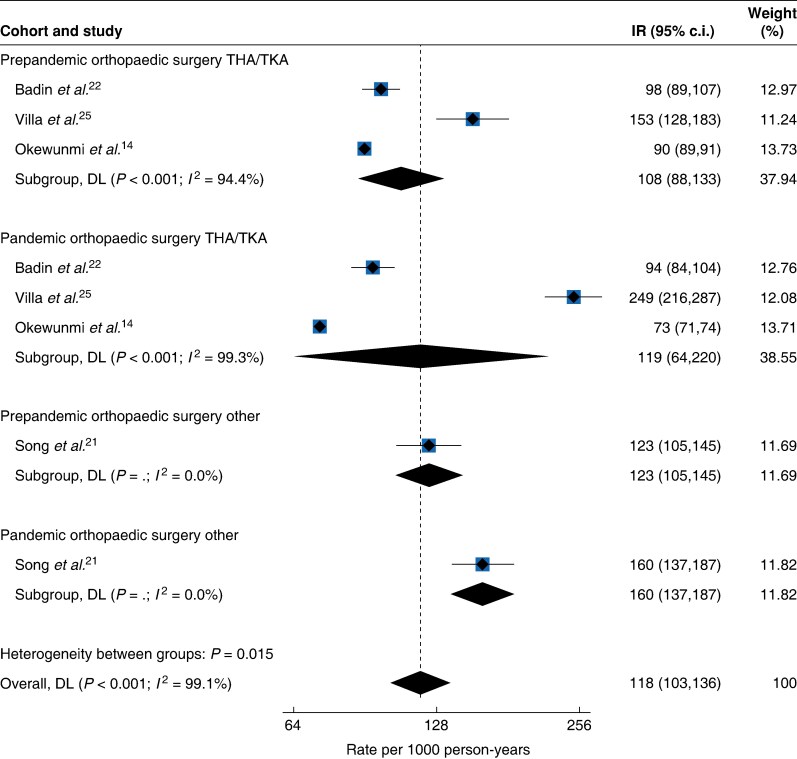
Postoperative VTE incidence rates per 1000 person-years after orthopaedic surgery according to pre-pandemic and pandemic intervals VTE, venous thromboembolism; IR, incidence rate; THA, total hip arthroplasty; TKA, total knee arthroplasty; DL, DerSimonian-Laird.

The aggregated absolute VTE risk among patients undergoing THA and TKA surgery was 2.2% (40 024 out of 1 862 002) during the pre-pandemic interval, compared with 1.7% (11 675 out of 682 525) in the pandemic interval, a 20% decrease in unadjusted relative risk. Additionally, the overall pooled incidence rate was 108 (95% c.i. 88 to 133, *I*^2^ = 94.4%) per 1000 person-years in the pre-pandemic interval, increasing to 119 (95% c.i. 64 to 220, *I*^2^ = 99.3%) per 1000 person-years during the pandemic (*Fig. 3*).

In the single study focusing on spinal surgery, a pre-pandemic absolute VTE risk of 1.0% (151 out of 14 973) was reported, compared with a pandemic absolute risk of 1.3% (163 out of 12 473), a 1.30-fold unadjusted relative risk increase, with the incidence rate increasing from 123 (95% c.i. 105 to 145) per 1000 person-years in the pre-pandemic interval to 160 (95% c.i. 137 to 187) per 1000 person-years during the pandemic (*Fig. 3*).

#### Frequency and pooled incidence of VTE events after EGS and multispecialty surgery according to COVID-19 infection status

**Fig. 4:**
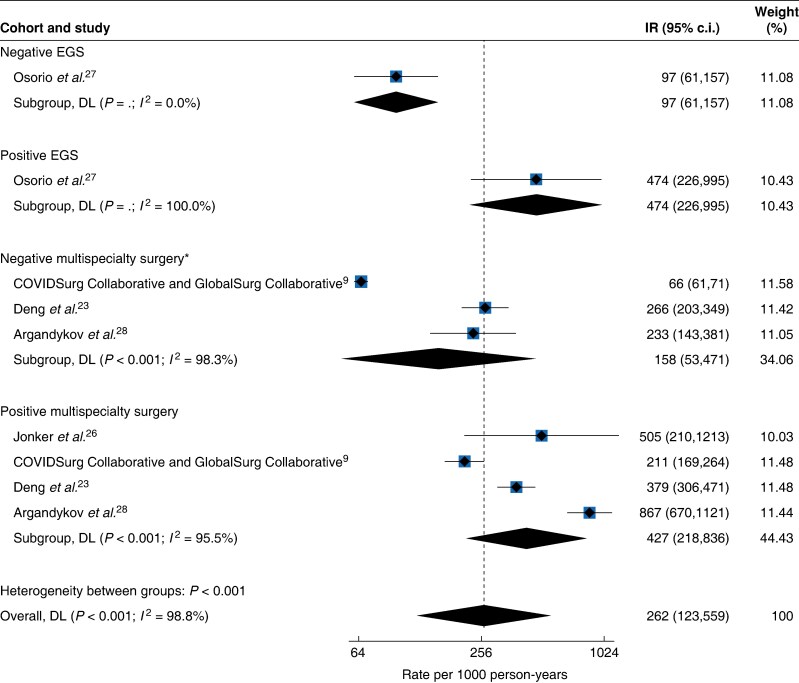
Postoperative VTE incidence rates per 1000 person-years after EGS and multispecialty surgery according to COVID-19 infection status *There were no VTE events in the Jonker *et al*.^26^ COVID-19-negative group. VTE, venous thromboembolism; EGS, emergency general and gastrointestinal surgery; IR, incidence rate; DL, DerSimonian-Laird.

A single study^27^ investigated the postoperative outcomes in patients undergoing EGS, focusing on those with and without perioperative COVID-19 infection (*Table 2*). It found an absolute VTE risk of 0.8% (17 out of 2132) in patients without COVID-19, compared with an absolute risk of 3.8% (7 out of 183) in COVID-19-infected patients, a 4.80-fold unadjusted relative risk increase. The incidence rate was 97 (95% c.i. 61 to 157) per 1000 person-years for non-infected patients, increasing to 474 (95% c.i. 226 to 995) per 1000 person-years for those infected with COVID-19 (*Fig. 4*).

Four additional studies^9,23,26,28^ included patients undergoing a variety of surgical procedures (*Table 2*). In these studies, the aggregated absolute VTE risk was 0.6% (735 out of 127 251) for COVID-19-negative patients and 2.7% (221 out of 8242) for COVID-19-positive patients, a 4.64-fold unadjusted relative risk increase. The pooled incidence rate was 158 (95% c.i. 53 to 471, *I*^2^ = 98.3%) per 1000 person-years for COVID-19-negative patients and 427 (95% c.i. 218 to 836, *I*^2^ = 95.5%) per 1000 person-years for COVID-19-positive patients (*Fig. 4*).

#### Frequency and pooled incidence of VTE events after EGS and multispecialty surgery comparing pre-pandemic and pandemic intervals

**Fig. 5:**
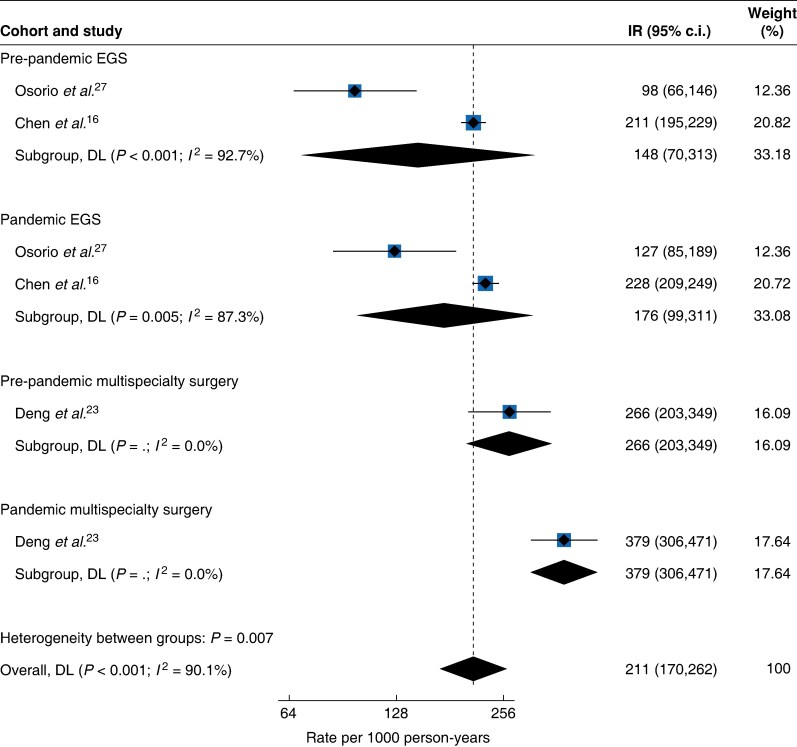
Postoperative VTE incidence rates per 1000 person-years after EGS and multispecialty surgery according to pre-pandemic and pandemic intervals VTE, venous thromboembolism; EGS, emergency general and gastrointestinal surgery; IR, incidence rate; DL, DerSimonian-Laird.

There were two studies that reported VTE events in the context of EGS during different pandemic intervals^16,27^, with an aggregated absolute VTE risk of 1.7% (623 out of 37 802) in the pre-pandemic interval, increasing to 1.8% (536 out of 29 898) during the pandemic, a 1.09-fold unadjusted relative risk increase (*Table 2*). The pooled incidence rates were 148 (95% c.i. 70 to 313, *I*^2^ = 92.7%) per 1000 person-years in the pre-pandemic interval and 176 (95% c.i. 99 to 311, *I*^2^ = 87.3%) per 1000 person-years in the pandemic interval (*Fig. 5*).

Additionally, a single study focusing on major elective surgeries^23^ within a specific patient cohort reported a 1.42-fold unadjusted relative risk increase in VTE events from the pre-pandemic interval to the pandemic interval (*Table 2*). The observed absolute VTE risk in the pre-pandemic cohort was 2.0% (53 out of 2621), rising to 2.9% (82 out of 2858) during the pandemic, resulting in incidence rates of 266 (95% c.i. 203 to 349) per 1000 person-years and 379 (95% c.i. 306 to 471) per 1000 person-years respectively (*Fig. 5*).

## Discussion

The findings of this study indicate a marked increase in VTE rates among COVID-19-positive patients, which was consistent in those undergoing orthopaedic surgery, major elective surgery, and EGS, with an almost five-fold unadjusted relative risk increase, compared with COVID-19-negative patients. However, the differences comparing pre-pandemic and pandemic intervals were less marked where COVID-19 exposures were less clearly documented.

The studies focusing solely on the pandemic interval in this review had similar baseline VTE risks in COVID-19-negative patients compared with pre-pandemic studies. This, combined with the smaller changes in the comparisons of pre-pandemic and pandemic intervals observed in this review, suggests that the studies within this analysis that focused on the pandemic interval included a similar surgical case mix to that before the COVID-19 pandemic. For example, the absolute aggregated VTE risks of 1.8% and 1.7% for orthopaedic THA/TKA COVID-19-negative and pandemic-interval patient cohorts respectively in this review compare favourably to the aggregated VTE risk of 1.9% observed in studies before the COVID-19 pandemic^29^.

Between pre-pandemic and pandemic intervals, globally there were slight increases in observed VTE incidence rates and unadjusted relative risks within pandemic-interval cohorts across orthopaedic surgery, EGS, and multispecialty surgery. Although, within the pandemic cohorts there were no overall clear definitions of COVID-19 exposure status, suggesting the observed minor increases in VTE rates may have been influenced by this, with the increases in VTE rates potentially attributable to perioperative COVID-19-positive status. Of note, the three orthopaedic THA/TKA studies that stratified patients into pre-pandemic and pandemic intervals^14,22,25^ saw a 20% reduction in aggregated unadjusted relative VTE risk from the pre-pandemic interval to the pandemic interval. However, when weighted for person-years, there was a slight increase in the observed pooled VTE incidence rate. This may relate to the largest study^14^ being the only THA/TKA study comparing pre-pandemic and pandemic intervals with a 90-day, as opposed to 30-day, follow-up that may have reduced the pooled incidence rate effect observed, as postoperative VTE incidence has been shown to be non-linear^30,31^. Also, Okewunmi *et al*.^14^ utilized a large pre-pandemic cohort dating back to the year 2016 when the VTE risk was higher (2.4%), which by 2019 had a similar pre-pandemic VTE risk (2.0%) to the THA/TKA COVID-19-negative patients seen in this study and so inflating the aggregated absolute THA/TKA pre-pandemic VTE risk seen in this review. In contrast, the spinal surgery VTE rates^21^ during the same intervals revealed a slight increase during the pandemic. However, these rates are not dissimilar to previous yearly VTE rates observed in lumbar surgery (1.1–1.2%)^32^.

In the emergency setting, this review included one general surgery study^27^, with an absolute postoperative VTE risk of 0.8% in their COVID-19-negative patients, consistent with that reported in the literature for overall general and colorectal surgery (0.9%) during the pandemic interval^9^. The largest difference in VTE rates by COVID-19 status was seen in this EGS subgroup, with an unadjusted relative risk increase of 4.80-fold. The high VTE rates in the COVID-19-positive group may very well reflect the pathophysiological effect of COVID-19 infection and increased VTE formation previously described^4,8^. Additionally, four other studies, covering multiple surgical specialties^9,23,26,28^, collectively reported an aggregated absolute VTE risk of 0.6% in COVID-19-negative patients. The stark differences in VTE risk by COVID-19 status between elective and emergency procedures highlights the need for further work in these areas. Particularly, as VTE rates appear to differ according to admission type, stratified risks are needed to better care for and council patients if the pandemic was to re-escalate.

This systematic review has demonstrated a consistent increased incidence of VTE among postoperative COVID-19-positive patients across various surgical specialties. Particularly, patients undergoing orthopaedic surgery, major elective surgery, and EGS exhibited an almost five-fold increased unadjusted relative risk of developing VTE compared with their COVID-19-negative counterparts.

These findings underscore the necessity for heightened vigilance and potentially revised VTE prophylaxis, including extended prophylaxis, strategies among clinicians treating perioperative patients with active or previous COVID-19 infection^33^. The significant increase in COVID-19-positive compared with COVID-19-negative rates of VTE across surgical specialties, in comparison with the minor non-significant increase in VTE rates seen between pre-pandemic and pandemic cohorts, suggests the case mix during the pandemic was comparable to pre-pandemic intervals. Current guidance cannot be changed based solely on this study; however, the findings highlight areas where perioperative intervention is needed to improve postoperative outcomes for patients. As the COVID-19 pandemic transitions to an endemic stage and surgical services are largely reinstated, the landscape for surgical care continues to evolve, particularly given that a significant proportion of the global population is now vaccinated^34^. This shift underscores the need for contemporary studies that not only encompass the initial phases of the pandemic but also span the entire course and so explore the protective nature of vaccination status, as well as the impact of COVID-19-variant differences and operation-specific differences (including sub-specialty type, urgency of surgery, and operative technique) on VTE risk. Specifically, further studies are needed to identify the risk of VTE in patients who are vaccinated against COVID-19 in relation to COVID-19 infection. Vaccination may not only affect the overall risk of developing COVID-19 but effect COVID-19 morbidity in relation to pro-inflammatory changes during infection and, as such, venous stasis and VTE risk^8^. This will enable more tailored guidance in appropriate prophylaxis and preventive strategies for clinicians going forward.

This meta-analysis has several strengths. An exhaustive list of five validated databases was utilized to ensure all relevant studies were included in the scope of the systematic review, although a language limitation was applied. Most studies within this meta-analysis were national/multicentre cohorts utilizing validated databases and outcome measures, as opposed to regional/single-centre studies, and so report truly representative population-based data, with a diverse global spread and pooled symptomatic VTE rates. Therefore, these data are likely to be more generalizable. Where possible, studies were consistently grouped by operation and surgical specialty, thus providing specific stratified VTE rates, as well as reducing the impact of known differences in VTE rates between specialties. However, there are several limitations.

Heterogeneity was >90% in most meta-analyses conducted. This is likely secondary to the use of large cohort studies reducing within-study variance and the inclusion of descriptive compared with comparative epidemiological studies where minor differences in population and outcome definitions, evident between the studies included in this review, can significantly affect incidence rates and the overall statistical estimate of a specified cohort, an effect previously reported^12,35^. Additionally, differences in the definitions of COVID-19 positivity and the exposure interval in relation to index surgery date between studies may affect inter-study estimates. Although all analyses were weighted based on the random-effects model to account for inter-study variance, they were all unadjusted for other potential confounding variables. Most studies that specifically examined patients diagnosed with COVID-19 did not specify whether patients were vaccinated against COVID-19 infection or whether patients identified as being positive for COVID-19 were symptomatic or asymptomatic, and so these subgroup analyses could not be performed. Thresholds for surgical intervention undoubtedly changed, especially during the early interval of the pandemic; however, the studies reporting on VTE risk before and during the pandemic did not fully report these differences. VTE risk is known to remain raised up to 3 months after surgery^31^ and, although several studies reported 90-day VTE, the majority only reported 30-day outcomes, potentially under-reporting the VTE rate. Due to the nature of and lack of data available within the included studies, it was not possible to fully stratify patients by admission type and operative technique, as initially intended, or to acutely examine differences in thromboprophylaxis regimens used after surgery and type of VTE (symptomatic *versus* asymptomatic), which may have influenced the overall rates observed. Yet, no specific VTE screening programmes were used within the studies suggesting most outcomes were symptomatic and international guidance during the pandemic advocating no change to current VTE prophylaxis regimens in non-clinically symptomatic hospitalized patients indicating likely no overall changes in thromboprophylaxis use occurred^36,37^. Over cohort time intervals, the effect of COVID-19 vaccination may have influenced the rates of VTE for patients operated on during the pandemic interval. However, most study intervals did not bridge large proportions of time either side of the initiation of national COVID-19 vaccination programmes between December 2020 and January 2021^38,39^. Most studies that did either excluded or accounted for those vaccinated or described it within their limitations. Despite including studies published in 2021 to 2024, the majority of studies did not examine time intervals later than 2021, meaning the effects of COVID-19 variants, changes in surgical pathways, and vaccination status on postoperative VTE could not be assessed.

## Supplementary Material

zraf039_Supplementary_Data

## Data Availability

Data are available upon reasonable request.
