# Supplementary material for: Venous thromboembolism risk in the postoperative interval during the COVID-19 pandemic: meta-analysis
Source: BJS Open. 2025 Apr 15;9(2):zraf039. doi: 10.1093/bjsopen/zraf039 (PMC11997968; doi:10.1093/bjsopen/zraf039)
Supplement: zraf039_Supplementary_Data [file zraf039_supplementary_data.docx]

**Venous Thromboembolism Risk in the Postoperative Period During the COVID-19 Pandemic: A Systematic Review and Meta-Analysis**

Andrew Jackson^1,2^, Christopher Anthony Lewis-Lloyd^1,2^ (ORCiD: 0000-0001-9996-3699), Oluwademilade Merotohun^1,2^, Colin John Crooks^1,2^ (ORCiD: 0000-0002-6794-6621) and David James Humes^1,2^ (ORCiD: 0000-0002-7071-4098)

^1^Nottingham Digestive Diseases Centre, Division of Translation Medical Sciences, School of Medicine, University of Nottingham, Queen's Medical Centre, Nottingham, UK.

^2^National Institute for Health Research (NIHR) Nottingham Biomedical Research Centre, Nottingham University Hospitals NHS Trust and University of Nottingham, Queen's Medical Centre, Nottingham, UK.

**Corresponding author.** Christopher Anthony Lewis-Lloyd, Nottingham Digestive Diseases Centre, Division of Translation Medical Sciences, School of Medicine, University of Nottingham, Queen's Medical Centre, Nottingham, UK, Derby Road, Nottingham, NG7 2UH, UK. **0000-0001-9996-3699**; **@ChrisLewisLloyd.**

**Supplementary Materials - Index**

| **Supplementary Figures and Tables** |  |
| --- | --- |
| Supplementary Figure 1: PRISMA Checklist | *page 2* |
| Supplementary Figure 2: MOOSE Checklist | *page 4* |
| Supplementary Figure 3: Search Strategies | *page 6* |
| Supplementary Table 1: Risk of Bias Assessment Table | *page 8* |
| Supplementary Figure 4: Risk of Bias Assessment Criteria | *page 9* |
| Supplementary Table 2: Description of studies for the qualitative analysis | *page 10* |
| **References** | *page 13* |

| **Section and Topic** | **Item #** | **Checklist item** | **Location where item is reported** |
| --- | --- | --- | --- |
| **TITLE** | | |  |
| Title | 1 | Identify the report as a systematic review. | 1 |
| **ABSTRACT** | | |  |
| Abstract | 2 | See the PRISMA 2020 for Abstracts checklist. | 3-4 |
| **INTRODUCTION** | | |  |
| Rationale | 3 | Describe the rationale for the review in the context of existing knowledge. | 5-6 |
| Objectives | 4 | Provide an explicit statement of the objective(s) or question(s) the review addresses. | 6 |
| **METHODS** | | |  |
| Eligibility criteria | 5 | Specify the inclusion and exclusion criteria for the review and how studies were grouped for the syntheses. | 7-8 |
| Information sources | 6 | Specify all databases, registers, websites, organisations, reference lists and other sources searched or consulted to identify studies. Specify the date when each source was last searched or consulted. | 7 |
| Search strategy | 7 | Present the full search strategies for all databases, registers and websites, including any filters and limits used. | 7 |
| Selection process | 8 | Specify the methods used to decide whether a study met the inclusion criteria of the review, including how many reviewers screened each record and each report retrieved, whether they worked independently, and if applicable, details of automation tools used in the process. | 8-9 |
| Data collection process | 9 | Specify the methods used to collect data from reports, including how many reviewers collected data from each report, whether they worked independently, any processes for obtaining or confirming data from study investigators, and if applicable, details of automation tools used in the process. | 8-9 |
| Data items | 10a | List and define all outcomes for which data were sought. Specify whether all results that were compatible with each outcome domain in each study were sought (e.g. for all measures, time points, analyses), and if not, the methods used to decide which results to collect. | 8-9 |
|  | 10b | List and define all other variables for which data were sought (e.g. participant and intervention characteristics, funding sources). Describe any assumptions made about any missing or unclear information. | 7-9 |
| Study risk of bias assessment | 11 | Specify the methods used to assess risk of bias in the included studies, including details of the tool(s) used, how many reviewers assessed each study and whether they worked independently, and if applicable, details of automation tools used in the process. | 9-10 |
| Effect measures | 12 | Specify for each outcome the effect measure(s) (e.g. risk ratio, mean difference) used in the synthesis or presentation of results. | 10 |
| Synthesis methods | 13a | Describe the processes used to decide which studies were eligible for each synthesis (e.g. tabulating the study intervention characteristics and comparing against the planned groups for each synthesis (item #5)). | 7-9 |
|  | 13b | Describe any methods required to prepare the data for presentation or synthesis, such as handling of missing summary statistics, or data conversions. | 10 |
|  | 13c | Describe any methods used to tabulate or visually display results of individual studies and syntheses. | 10 |
|  | 13d | Describe any methods used to synthesize results and provide a rationale for the choice(s). If meta-analysis was performed, describe the model(s), method(s) to identify the presence and extent of statistical heterogeneity, and software package(s) used. | 10 |
|  | 13e | Describe any methods used to explore possible causes of heterogeneity among study results (e.g. subgroup analysis, meta-regression). | N/A |
|  | 13f | Describe any sensitivity analyses conducted to assess robustness of the synthesized results. | N/A |
| Reporting bias assessment | 14 | Describe any methods used to assess risk of bias due to missing results in a synthesis (arising from reporting biases). | 9-10, Table S1, Figure S4 |
| Certainty assessment | 15 | Describe any methods used to assess certainty (or confidence) in the body of evidence for an outcome. | N/A |
| **RESULTS** | | |  |
| Study selection | 16a | Describe the results of the search and selection process, from the number of records identified in the search to the number of studies included in the review, ideally using a flow diagram. | 12-13 |
|  | 16b | Cite studies that might appear to meet the inclusion criteria, but which were excluded, and explain why they were excluded. | 12, Figure 1 |
| Study characteristics | 17 | Cite each included study and present its characteristics. | 12-13, Tables 1-2 |
| Risk of bias in studies | 18 | Present assessments of risk of bias for each included study. | Table S1, Figure S4 |
| Results of individual studies | 19 | For all outcomes, present, for each study: (a) summary statistics for each group (where appropriate) and (b) an effect estimate and its precision (e.g. confidence/credible interval), ideally using structured tables or plots. | 13-16 |
| Results of syntheses | 20a | For each synthesis, briefly summarise the characteristics and risk of bias among contributing studies. | 13-16 |
|  | 20b | Present results of all statistical syntheses conducted. If meta-analysis was done, present for each the summary estimate and its precision (e.g. confidence/credible interval) and measures of statistical heterogeneity. If comparing groups, describe the direction of the effect. | 13-16, Figures 2-5 |
|  | 20c | Present results of all investigations of possible causes of heterogeneity among study results. | 13-16 |
|  | 20d | Present results of all sensitivity analyses conducted to assess the robustness of the synthesized results. | N/A |
| Reporting biases | 21 | Present assessments of risk of bias due to missing results (arising from reporting biases) for each synthesis assessed. | Table S1, Figure S4 |
| Certainty of evidence | 22 | Present assessments of certainty (or confidence) in the body of evidence for each outcome assessed. | N/A |
| **DISCUSSION** | | |  |
| Discussion | 23a | Provide a general interpretation of the results in the context of other evidence. | 17-19 |
|  | 23b | Discuss any limitations of the evidence included in the review. | 19-20 |
|  | 23c | Discuss any limitations of the review processes used. | 19-20 |
|  | 23d | Discuss implications of the results for practice, policy, and future research. | 20-21 |
| **OTHER INFORMATION** | | |  |
| Registration and protocol | 24a | Provide registration information for the review, including register name and registration number, or state that the review was not registered. | 1-2 |
|  | 24b | Indicate where the review protocol can be accessed, or state that a protocol was not prepared. | 7 |
|  | 24c | Describe and explain any amendments to information provided at registration or in the protocol. | N/A |
| Support | 25 | Describe sources of financial or non-financial support for the review, and the role of the funders or sponsors in the review. | 1-2 |
| Competing interests | 26 | Declare any competing interests of review authors. | 1-2 |
| Availability of data, code and other materials | 27 | Report which of the following are publicly available and where they can be found: template data collection forms; data extracted from included studies; data used for all analyses; analytic code; any other materials used in the review. | 1-2 |

*From:*  Page MJ, McKenzie JE, Bossuyt PM, Boutron I, Hoffmann TC, Mulrow CD, et al. The PRISMA 2020 statement: an updated guideline for reporting systematic reviews. BMJ 2021;372:n71. doi: 10.1136/bmj.n71

Supplementary Figure 2: MOOSE (Meta-analyses Of Observational Studies in Epidemiology) Checklist

| **Reporting Criteria** | **Reported (Yes/No)** | **Reported on Page No.** |
| --- | --- | --- |
| **Reporting of Background** | Yes | 5-6 |
| Problem definition | Yes | 5-6 |
| Hypothesis statement | No |  |
| Description of Study Outcome(s) | Yes | 10 |
| Type of exposure or intervention used | Yes | 6-9 |
| Type of study design used | Yes | 7-9 |
| Study population | Yes | 7-9 |
| **Reporting of Search Strategy** | Yes | 7, Figure S3 |
| Qualifications of searchers (e.g., librarians and investigators) | Yes | 1 |
| Search strategy, including time period included in the synthesis and keywords | Yes | 7, Figure S3 |
| Effort to include all available studies, including contact with authors | Yes | 7 |
| Databases and registries searched | Yes | 7, Figure S3 |
| Search software used, name and version, including special features used (e.g., explosion) | Yes | 7, Figure S3 |
| Use of hand searching (e.g., reference lists of obtained articles) | Yes | 7, Figure S3 |
| List of citations located and those excluded, including justification | Yes | Figure 1 |
| Method for addressing articles published in languages other than English | Yes | 7 |
| Method of handling abstracts and unpublished studies | Yes | Figure 1 |
| Description of any contact with authors | No |  |
| **Reporting of Methods** | Yes | 7-10 |
| Description of relevance or appropriateness of studies assembled for assessing the hypothesis to be tested | Yes | 7-10, Figure 1 |
| Rationale for the selection and coding of data (e.g., sound clinical principles or convenience) | Yes | 9-10, Table S1, Figure S4 |
| Documentation of how data were classified and coded (e.g., multiple raters, blinding, and interrater reliability) | Yes | 7-9 |
| Assessment of confounding (e.g., comparability of cases and controls in studies where appropriate | No |  |
| Assessment of study quality, including blinding of quality assessors; stratification or regression on possible predictors of study results | Yes | Table S1, Figure S4 |
| Assessment of heterogeneity | No |  |
| Description of statistical methods (e.g., complete description of fixed or random effects models, justification of whether the chosen models account for predictors of study results, dose-response models, or cumulative meta-analysis) in sufficient detail to be replicated | Yes | 10 |
| Provision of appropriate tables and graphics | Yes | 10, Tables 1-2, Figures 2-5 |
| **Reporting Criteria** | **Reported (Yes/No)** | **Reported on Page No.** |
| **Reporting of Results** | Yes | 12-16 |
| Table giving descriptive information for each study included | Yes | 12-13, Tables 1-2 |
| Results of sensitivity testing (e.g., subgroup analysis) | No |  |
| Indication of statistical uncertainty of findings | Yes | 13-16 |
| **Reporting of Discussion** | Yes | 17-21 |
| Quantitative assessment of bias (e.g., publication bias) | Yes | Table S1, Figure S4 |
| Justification for exclusion (e.g., exclusion of non–English-language citations) | Yes | 7 |
| Assessment of quality of included studies | Yes | 19-20, Table S1, Figure S4 |
| **Reporting of Conclusions** | Yes | 20-21 |
| Consideration of alternative explanations for observed results | Yes | 17-21 |
| Generalization of the conclusions (i.e., appropriate for the data presented and within the domain of the literature review) | Yes | 20-21 |
| Guidelines for future research | Yes | 21 |
| Disclosure of funding source | Yes | 1-2 |

Supplementary Figure 3: Search Strategies

| **#** | **Query** | **Results from 6 Nov 2023** |
| --- | --- | --- |
| 1 | exp COVID-19/ or exp coronavirus disease 2019/ or exp Coronavirus infection/ or exp Coronavirus infection/ or exp coronavirus disease 2019/ or "2019-nCoV Infection".mp. or "SARS-CoV-2 Infection".mp. or "COVID-19 Virus Infection".mp. or "2019 Novel Coronavirus Disease".mp. or "2019 Novel Coronavirus Infection".mp. or "Severe Acute Respiratory Syndrome Coronavirus 2 Infection".mp. or "COVID19".mp. or "Coronavirus Disease 2019".mp. or "2019-nCoV Disease".mp. or "COVID-19 Pandemic".mp. or "SARS Coronavirus 2 Infection".mp. or Covid-19.mp. or Coronavirus Infections.mp. or coronavirus-19.mp. | 458,436 |
| 2 | ("2019-nCoV*" or 2019nCoV* or "19-nCoV*" or 19nCoV* or nCoV2019* or "nCoV-2019*" or nCoV19* or "nCoV19*" or ("COVID-19*" or COVID19* or "COVID2019*" or covid) or ("HCoV-19*" or HCoV19* or "HCoV-2019*" or HCoV2019*) or ("2019 novel*" or Ncov* or "ncov") or ("SARS-CoV-2*" or "SARSCoV-2*" or "SARSCoV2*" or "SARS-CoV2*") or (SARSCov19* or "SARS-Cov19*" or "SARSCov-19*" or "SARS-Cov-19*" or SARSCov2019* or "SARS-Cov2019*" or "SARSCov-2019*" or "SARS-Cov-2019*") or (SARS2* or "SARS-2*") or (SARScoronavirus2* or "SARS-coronavirus-2*") or (respiratory* adj2 (symptom* or disease* or illness* or condition*) adj5 (Wuhan* or Hubei* or China* or Chinese* or Huanan*)) or (("seafood market*" or "food market*") adj10 (Wuhan* or Hubei* or China* or Chinese* or Huanan*)) or (pneumonia* adj3 (Wuhan* or Hubei* or China* or Chinese* or Huanan*)) or ((outbreak* or wildlife* or pandemic* or epidemic*) adj1 (Wuhan* or Hubei* or China* or Chinese* or Huanan*)) or "severe acute respiratory syndrome*").ti,ab,kw. | 432,443 |
| 3 | 1 or 2 | 478,241 |
| 4 | (surg* or operat* or surger* or procedur* or (ectomy or otomy or section)).mp. or exp Surgical Procedures, Operative/ or exp Laparoscopy/ or exp Robotic surgical procedures/ | 9,735,023 |
| 5 | (VTE or DVT or PE or (thromb* adj2 (vein* or venous)) or (venous adj stasis) or venous thromboembolism).mp. or exp venous thromboembolism/ or exp thromboembolism/ or exp lung embolism/ or exp vein thrombosis/ or (thromboembolism or venous thromb* or venous embol*).mp. or (deep vein thromb* or pulmonary embol* or lung embol*).mp. or (phlegmasia adj2 dolens).mp. or paradoxical embolism.mp. or exp paradoxical embolism/ or ((pulmonary or lung) adj4 (embol* or thromboembo* or microembol*)).mp. or (pulmonary adj infarction).mp. | 815,492 |
| 6 | 3 and 4 and 5 | 7,087 |
| 7 | exp Case reports/ or Case report.tw. or exp abstract report/ or exp letter/ or conference proceeding.pt. or conference abstract.pt. or editorial.pt. or letter.pt. or note.pt. | 8,563,975 |
| 8 | 6 not 7 | 4,024 |
| 9 | limit 8 to (english language and yr="2019 -Current") | 3,891 |

**Database:** Embase <1974 to 2023 November 03>

**Database:** Ovid MEDLINE(R) ALL <1946 to November 03, 2023>

| **#** | **Query** | **Results from 6 Nov 2023** |
| --- | --- | --- |
| 1 | ("2019-nCoV*" or 2019nCoV* or "19-nCoV*" or 19nCoV* or nCoV2019* or "nCoV-2019*" or nCoV19* or "nCoV-19*" or "COVID-19*" or COVID19* or "COVID-2019*" or covid or "HCoV-19*" or HCoV19* or "HCoV-2019*" or HCoV2019 or "2019 novel*" or Ncov* or "n-cov" or "SARS-CoV-2*" or "SARSCoV-2*" or "SARSCoV2*" or "SARS-CoV2*" or SARSCov19* or "SARS-Cov19*" or "SARSCov-19*" or "SARS-Cov-19*" or SARSCov2019* or "SARS-Cov2019*" or "SARSCov-2019*" or "SARS-Cov-2019*" or SARS2* or "SARS-2*" or SARScoronavirus2* or "SARS-coronavirus-2*" or "severe acute respiratory syndrome").mp. or exp Pulmonary Embolism/ or exp Severe Acute Respiratory Syndrome/ or exp COVID-19/ or exp Coronavirus Infections/ | 447,121 |
| 2 | exp specialties, surgical/ or exp Surgical Procedures, Operative/ or exp Laparoscopy/ or exp Robotic surgical procedures/ or (surg* or operat* or surger* or procedur*).mp. or (ectomy or otomy or section).mp. | 6,716,458 |
| 3 | (VTE or DVT or PE or (thromb* adj2 (vein* or venous)) or (venous adj stasis) or venous thromboembolism or (thromboembolism or venous thromb* or venous embol*) or (deep vein thromb* or pulmonary embol* or lung embol*) or (phlegmasia adj2 dolens) or paradoxical embolism or ((pulmonary or lung) adj4 (embol* or thromboembo* or microembol*)) or (pulmonary adj infarction)).mp. or exp Venous thromboembolism/ or exp Thromboembolism/ or exp Pulmonary embolism/ or exp Venous thrombosis/ or exp Embolism, Paradoxical/ | 270,474 |
| 4 | 1 and 2 and 3 | 16,409 |
| 5 | exp Case reports/ or exp Historical article/ or exp Letter/ | 3,773,389 |
| 6 | 4 not 5 | 11,131 |
| 7 | limit 6 to (english language and yr="2019 -Current") | 2,035 |

**Database:** Cochrane Library

| ID | Search Hits |  |
| --- | --- | --- |
| #1 | MeSH descriptor: [COVID-19] explode all trees | 4894 |
| #2 | Coronavirus | 10663 |
| #3 | #1 or #3 | 12756 |
| #4 | MeSH descriptor: [Specialties, Surgical] explode all trees | 3728 |
| #5 | Surgery | 304618 |
| #6 | #4 or #5 | 305935 |
| #7 | MeSH descriptor: [Venous Thromboembolism] explode all trees | 1342 |
| #8 | MeSH descriptor: [Venous Thrombosis] explode all trees | 3286 |
| #9 | (venous thromb*):ti,ab,kw (Word variations have been searched) | 10732 |
| #10 | #7 or #8 or #9 | 11752 |
| #11 | #3 and #6 and #10 | 20 |
| #12 | #3 and #6 and #10 with Publication Year from 2019 to 2023, with Cochrane Library publication date Between Jan 2019 and Nov 2023, in Trials | 17 |

Supplementary Table 1: Risk of Bias Assessment Table (Modified Newcastle Ottawa Scale)

| **Study**  **(Year)** | **Selection** | | | | **Comparability** | **Outcome** | | | **Total**  **(13 *)** | **Overall risk of bias**  **(Low, Moderate, High)** |
| --- | --- | --- | --- | --- | --- | --- | --- | --- | --- | --- |
|  | **Representativeness of exposed cohort**  **(* *)** | **Selection of non-exposed cohort**  **(*)** | **Exposure ascertainment**  **(* *)** | **Outcome of interest not present at study of start**  **(*)** | **Study contains matched cohorts for risk factors**  **(* *)** | **Outcome assessment**  **(* *)** | **Follow-up length**  **(*)** | **Follow-up adequacy**  **(* *)** |  |  |
| Badin  (2022) (1) | *** *** | ***** | *** *** | **-** | ***** | ***** | ***** | ***** | **9 *** | **Moderate** |
| Forlenza  (2022) (2) | ***** | ***** | *** *** | **-** | ***** | ***** | ***** | ***** | **8 *** | **Moderate** |
| Heckmann  (2023) (3) | *** *** | ***** | *** *** | **-** | *** *** | ***** | ***** | ***** | **10 *** | **Low** |
| Heo  (2024) (4) | ***** | ***** | *** *** | **-** | *** *** | ***** | ***** | ***** | **9 *** | **Moderate** |
| Lee  (2023) (5) | *** *** | ***** | *** *** | **-** | *** *** | ***** | ***** | ***** | **10 *** | **Low** |
| Okewunmi  (2024) (6) | *** *** | ***** | ***** | **-** | ***** | *** *** | ***** | ***** | **9 *** | **Moderate** |
| Villa  (2022) (7) | *** *** | ***** | *** *** | **-** | ***** | *** *** | ***** | ***** | **10 *** | **Low** |
| Wenzel  (2024) (8) | *** *** | ***** | *** *** | **-** | *** *** | ***** | ***** | ***** | **10 *** | **Low** |
| Johnson  (2023) (9) | *** *** | ***** | *** *** | **-** | *** *** | *** *** | ***** | ***** | **11 *** | **Low** |
| Mercier  (2023)(10) | *** *** | ***** | *** *** | **-** | ***** | *** *** | ***** | ***** | **10 *** | **Low** |
| Song  (2023) (11) | *** *** | ***** | *** *** | **-** | ***** | ***** | ***** | ***** | **9 *** | **Moderate** |
| Chen  (2023) (12) | *** *** | ***** | *** *** | **-** | ***** | *** *** | ***** | ***** | **10 *** | **Low** |
| Osorio  (2021) (13) | *** *** | ***** | *** *** | **-** | ***** | *** *** | ***** | *** *** | **11 *** | **Low** |
| Argandykov  (2023) (14) | *** *** | **-** | ***** | **-** | *** *** | ***** | ***** | ***** | **8 *** | **Moderate** |
| COVIDSurg Collaborative  (2022) (15) | *** *** | ***** | ***** | **-** | ***** | ***** | ***** | *** *** | **9 *** | **Moderate** |
| Deng  (2022) (16) | ***** | **-** | *** *** | **-** | **-** | ***** | ***** | ***** | **6 *** | **Moderate** |
| Jonker  (2021) (17) | *** *** | ***** | *** *** | **-** | *** *** | ***** | ***** | *** *** | **11 *** | **Low** |

Supplementary Figure 4: Risk of Bias Assessment Criteria (Modified Newcastle Ottawa Scale)


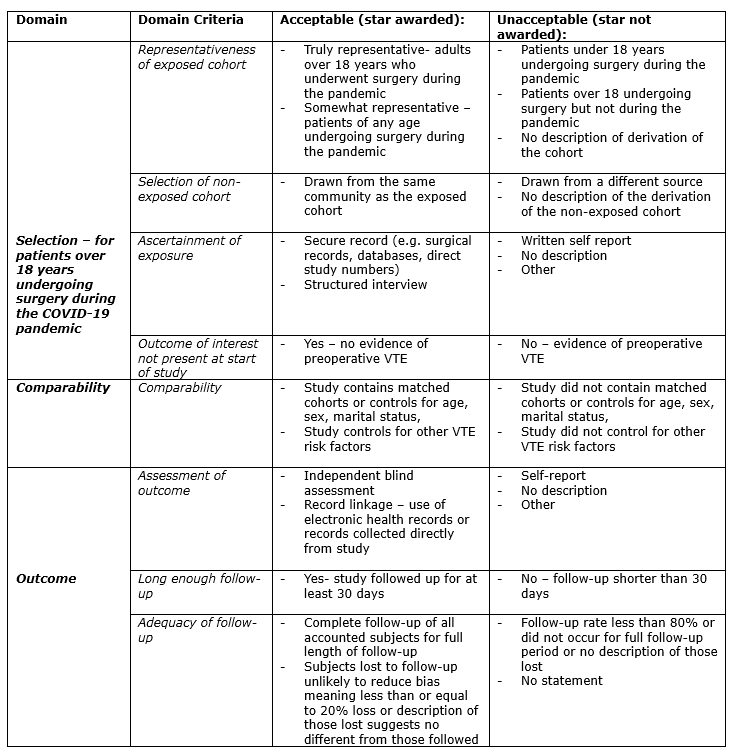

***** Note: A study can be given a maximum of one star for each numbered item within the Selection and Outcome categories. A maximum of two stars can be given for Comparability

| **Author**  **(Year)** | **Data source**  **(Country)** | **Follow up** | **Study time frame** | **Surgical procedures** | **VTE type** | **Total population** | **Overall No. of VTE events**  **(VTE incidence risk %)** | **Cohort definition** | |
| --- | --- | --- | --- | --- | --- | --- | --- | --- | --- |
|  |  |  |  |  |  |  |  | **Phase** | **SARS-CoV-2 positivity** |
| **Description of orthopaedic total knee & total hip arthroplasty studies** | | | | | | | | | |
| Badin  (2022) (1) | ACS-NSQIP  (USA & Canada) | 30 days | Jul-Dec 2019 & Jul-Dec 2020 | THA & TKA | DVT & PE | 100180 | 787  (0.79%) | Prepandemic: Jul-Dec 2019  Pandemic: Jul-Dec 2020 |  |
| Forlenza  (2022) (2) | Mariner & PearlDiver  (USA) | 90 days | Jan 2018-Apr 2020 | THA & TKA | DVT & PE | 5680 | 229  (4.03%) |  | ≤90 days postoperatively  ICD-10 D-U07.1 code |
| Heckmann  (2023) (3) | Premier Healthcare Database  (USA) | 90 days | Jan-Dec 2020 | THA & TKA | DVT & PE | 4984 | 14  (0.28%) |  | ±90 days perioperatively  ICD-10 U07.1 code |
| Heo  (2023) (4) | IBM MarketScan & Medicare  (USA) | 90 days | Jan 2018-Dec 2021 | Revision THA & TKA | DVT & PE | 660 | 24  (3.64%) |  | ≤90 days postoperatively  ICD-10 U071 & J12.82 codes |
| Lee  (2023) (5) | Mariner & PearlDiver  (USA) | 90 days | Jan 2020-Jan 2021 | THA & TKA | DVT & PE | 7230 | 220  (3.04%) |  | ≤90 days preoperatively  ICD-10-D-U071 code |
| Okewunmi  (2023) (6) | Medicare  (USA) | 90 days | Jan 2016-Sep 2021 | THA & TKA | DVT, PE & VTE | 2422051 | 50600  (2.09%) | Prepandemic: Jan 2016-Jan 2020  Pandemic: Feb 2020-Sep 2021 (includes post vaccine period) | Prior history of SARS-Cov-2  ICD-10 U10, U08, U09, U071, U099, & Z86.16 codes |
| Villa  (2022) (7) | Cleveland Clinic Florida  (USA) | 30 days | Dec 2018-Mar 2021 | THA & TKA | VTE | 19068 | 312  (1.64%) | Prepandemic: Dec 2018-Dec 2019  Pandemic: Jan 2020-Mar 2021 |  |
| Wenzel  (2023) (8) | ACS-NSQIP  (USA & Canada) | 30 days | Jan-Dec 2021 | THA & TKA | DVT & PE | 2340 | 36  (1.54%) |  | ≤30 days postoperatively  ICD-10 U07.1 code (U07.2 excluded) |
| **Description of other orthopaedic surgery studies** | | | | | | | | | |
| Johnson  (2023) (9) | TriNetX Research Network  (International) | 90 days | Apr 2020-Jan 2022 | Arthroscopic, total joint arthroplasty, lumbar fusion, upper extremity, foot & ankle | VTE | 48084 | 2486  (5.17%) |  | 7-90 days preoperatively  ICD-10 U07.1-2 & J12.82 codes |
| Mercier  (2023) (10) | ACS-NSQIP  (USA & Canada) | 30 days | Jan-Dec 2021 | Total joint arthroplasty, trauma surgery, spinal surgery, sports surgery & shoulder surgery | VTE | 194121 | 1710  (0.88%) |  | ≤14 days preoperatively  ICD-10 U07.1 code |
| Song  (2023) (11) | ACS-NSQIP  (USA & Canada) | 30 days | 2019-2020 | Lumbar fusion | DVT & PE | 27446 | 314  (1.14%) | Prepandemic: 2019  Pandemic: 2020 |  |
| **Description of emergency gastrointestinal & general surgery studies** | | | | | | | | | |
| Chen  (2022) (12) | ACS-NSQIP  (USA & Canada) | 30 days | Apr 2019-Dec 2020 | Colorectal, abdominoperineal resection, low anterior resection, total proctocolectomy & ostomy creation/revision | VTE | 62393 | 1111  (1.78%) | Prepandemic: Apr-Dec 2019  Pandemic: Apr-Dec 2020 |  |
| Osorio  (2021) (13) | COVID-CIR multicentre cohort study  (Spain) | 30 days | Mar-Jun 2019 & Mar-Jun 2020 | Perianal surgery, hernia/ eventration repair, appendectomy, cholecystectomy, colectomy, intestinal resection, lysis of adhesions or internal hernia repair, gastrointestinal perforation suture, splenectomy, minor liver trauma, gastrectomy, biliary surgery, exploratory laparotomy, other surgery, damage control surgery, pancreatectomy, pancreatic necrosectomy & emergency laparotomy | VTE | 5307 | 48  (0.90%) | Prepandemic: Mar-Jun 2019  Pandemic: Mar-Jun 2020 | ≤15 days preoperatively to 30 days postoperatively  RT-PCR or clinical diagnosis with CT positivity entry |
| **Description of multispecialty surgery studies** | | | | | | | | | |
| Argandykov  (2023) (14) | ACS-NSQIP 2012 cohort & COVIDSurg multicentre cohort study  (USA only) | 30 days | Feb-Jul 2020 | Neurosurgery, general surgery, thoracic, plastics, gynaecology, ENT surgery, cardiac surgery, urology, orthopaedics and vascular surgery | DVT & PE | 1686 | 74  (4.39%) |  | ≤7 days preoperatively to 30 days postoperatively  RT-PCR, Rapid antigen, immunoglobulin, CT, clinical diagnosis positive entry |
| COVIDSurg Collaborative  (2022) (15) | COVIDSurg multicentre cohort study  (International) | 30 days | Oct-Nov 2020 | Neuro, thoracic, colorectal, oncology, orthopaedic, spinal, cardiac, HPB, ENT, gynaecology, obstetric, vascular, general, hernia, oesophagogastric, plastic, other, dental, breast, endocrine surgery &, ophthalmology | VTE | 128009 | 742  (0.58%) |  | Perioperative: ≤7 days preoperatively to 30 days post operatively  Recent: 7-42 days preoperatively  Previous: >42 days preoperatively  RT-PCR, Rapid antigen, immunoglobulin, CT, clinical diagnosis positive entry |
| Deng  (2022) (16) | Symphony Health COVID-19 Research  (USA) | 30 days | May 2019-May 2021 | AAA repair (open/ endovascular), CABG, CEA, colonic surgery, oesophagogastric, liver resection, hip and knee arthroplasty, laminectomy, spinal arthrodesis, hysterectomy, lung resection, neurosurgery, pancreatic resection & prostatectomy | DVT & PE | 5479 | 135  (2.46%) | Prepandemic: May 2019-Jan 2020  Pandemic: Mar 2020-May 2021 (all SARS-Cov-2 positive) | Perioperative:  0-4 weeks postoperatively  Early:  4-8 weeks postoperatively  Late:  >8 weeks postoperatively  ICD-10 U07.1 code |
| Jonker  (2020) (17) | National multicentre cohort study  (Netherlands) | 30 days | Feb-Jun 2020 | Wall exploration, adnexal surgery, adrenalectomy, lower limb amputation, appendectomy, arthrotomy axillary lymph node dissection, brachiocephalic fistula, neck dissection, cholecystectomy, colectomy, escharotomy, melanoma excision, external fixation lower and upper extremity, gastrectomy, gastroschisis repair, gastrostomy, hysterectomy, Ileocecal resection, inguinal hernia repair, inguinal lymph node dissection, intramedullary nailing proximal femur, intramedullary nailing tibia, biliary surgery, kidney transplantation, knee ligament repair, laparoscopy, laparotomy, liver resection, liver transplantation, lobectomy, lymph node biopsy, mastectomy/ lumpectomy, mediastinoscopy, nephrectomy, oesophageal resection, orchiectomy, ORIF–femoral neck, pancreatic resection, parathyroidectomy, perianal abscess/ fistula, permanent dialysis catheter insertion, placenta removal, pyloromyotomy, radiological drainage, rectopexy, small bowel resection, resection soft tissue tumour, caesarean section, sigmoid resection, splenectomy, tracheotomy, trauma, vascular bypass, VATS empyema drainage, VATS pleurectomy, venous access port placement & wedge resection | DVT & PE | 319 | 5  (1.57%) |  | ≤30 days preoperatively to ≤30 days postoperatively  RT-PCR or clinical diagnosis with CT positivity entry |
| The Cardiothoracic Interdisciplinary Research Network  (2021) (18) | COVIDSurg multicentre cohort study  (International) | 30 days | Mar-Jul 2020 | Cardiac surgery | DVT & PE | 207  (all SARS-Cov-2 positive) | 8  (3.86%) |  | ≤7 days preoperatively to 30 days postoperatively  RT-PCR, Rapid antigen, immunoglobulin, CT, clinical diagnosis positive entry |

Supplementary Table 2: Description of studies for the qualitative analysis

AAA, Abdominal aortic aneurysm; ACS-NSQIP, American College of Surgeons National Surgical Quality Improvement Programme; CABG, Coronary artery bypass graft; CEA, Carotid endarterectomy; CT, Computed tomography; DVT, Deep vein thrombosis; ENT, Ear, Nose & Throat; HPB, Hepatopancreaticobiliary; ICD-10, International Classification of Diseases Tenth Revision; IBM, International Business Machines; ORIF, Open reduction internal fixation; PE, Pulmonary embolism; RT-PCR, Reverse transcription polymerase chain reaction; SARS-CoV-2, Severe Acute Respiratory Syndrome Coronavirus-2; THA & TKA, Total hip & knee arthroplasty; USA, United States of America; VATS, Video-assisted thoracoscopic surgery; VTE, Venous thromboembolism

ICD-10 codes: J12.82; Pneumonia due to coronavirus disease 2019. U07.1; COVID-19, virus identified (confirmed by laboratory testing). U07.2; COVID-19, virus not identified (diagnosed clinically or epidemiologically). U08; Personal history of COVID-19. U09; Post COVID-19 condition. U09.9; Post COVID-19 condition, unspecified. U10; Multisystem inflammatory syndrome associated with COVID-19. Z86.16; Personal history of infectious and parasitic diseases (Personal history of COVID-19)

**References**

1. Badin D, Ortiz-Babilonia CD, Harris AB, Raad M, Oni JK. Early Postoperative Complications in Total Hip and Knee Arthroplasties Before and During the COVID-19 Pandemic: A Retrospective Analysis of 38,234 Patients. Arthroplast Today. 2022;18:24-30.

2. Forlenza EM, Higgins JDD, Burnett RA, Serino J, Della Valle CJ. COVID-19 Infection After Total Joint Arthroplasty Is Associated With Increased Complications. J Arthroplasty. 2022;37(7S):S457-S64.

3. Heckmann ND, Wang JC, Piple AS, Bouz GJ, Chung BC, Oakes DA, et al. Positive COVID-19 Diagnosis Following Primary Elective Total Joint Arthroplasty: Increased Complication and Mortality Rates. J Arthroplasty. 2023;38(9):1682-92 e2.

4. Heo KY, Bonsu JM, Muffly BT, Rieger E, Song J, Ayeni AM, et al. Complications Rates Among Revision Total Knee Arthroplasty Patients Diagnosed With COVID-19 Postoperatively. J Arthroplasty. 2024;39(3):766-71 e2.

5. Lee A, Durst CR, Rezzadeh KT, Rajaee SS, Penenberg BL, Than JP. Higher Complication Rate in COVID-19 Recovered Patients Undergoing Primary Total Joint Arthroplasty. J Arthroplasty. 2023;38(7 Suppl 2):S111-S5.

6. Okewunmi JO, Ren R, Zubizarreta N, Kodali H, Poeran J, Hayden BL, et al. Prior COVID-19 and Venous Thromboembolism Risk in Total Joint Arthroplasty in Patients Over 65 Years of Age. J Arthroplasty. 2024;39(3):819-24 e1.

7. Villa JM, Pannu TS, Piuzzi NS, Krebs V, Riesgo AM, Higuera CA. A major increase of thromboembolic events in total hip and knee arthroplasty patients during the COVID-19 pandemic. Hosp Pract. 2022;50(1):68-74.

8. Wenzel AN, Marrache M, Schmerler J, Kinney J, Khanuja HS, Hegde V. Impact of Postoperative COVID-19 Infection Status on Outcomes in Elective Primary Total Joint Arthroplasty. J Arthroplasty. 2024;39(4):871-7.

9. Johnson AH, Stock LA, Petre BM, Keblish DJ, Gelfand J, Patton CM, et al. Postoperative Outcomes in Patients Undergoing Orthopaedic Surgery Within 90 Days of Coronavirus Disease 2019. J Am Acad Orthop Surg. 2023;31(3):148-54.

10. Mercier MR, Koucheki R, Lex JR, Khoshbin A, Park SS, Daniels TR, et al. The association between preoperative COVID-19-positivity and acute postoperative complication risk among patients undergoing orthopedic surgery. Bone Jt Open. 2023;4(9):704-12.

11. Song J, Katz AD, Qureshi SA, Virk SS, Sarwahi V, Silber J, et al. Lumbar fusion during the COVID-19 pandemic: greater rates of morbidity and longer procedures. J Spine Surg. 2023;9(1):73-82.

12. Chen SY, Radomski SN, Stem M, Papanikolaou A, Gabre-Kidan A, Atallah C, et al. Colorectal Surgery Outcomes in the United States During the COVID-19 Pandemic. J Surg Res. 2023;287:95-106.

13. Osorio J, Madrazo Z, Videla S, Sainz B, Rodriguez-Gonzalez A, Campos A, et al. Analysis of outcomes of emergency general and gastrointestinal surgery during the COVID-19 pandemic. Br J Surg. 2021;108(12):1438-47.

14. Argandykov D, Dorken-Gallastegi A, El Moheb M, Gebran A, Proano-Zamudio JA, Bokenkamp M, et al. Is perioperative COVID-19 really associated with worse surgical outcomes? A nationwide COVIDSurg propensity-matched analysis. J Trauma Acute Care Surg. 2023;94(4):513-24.

15. COVIDSurg Collaborative, GlobalSurg Collaborative. SARS-CoV-2 infection and venous thromboembolism after surgery: an international prospective cohort study. Anaesthesia. 2022;77(1):28-39.

16. Deng JZ, Chan JS, Potter AL, Chen YW, Sandhu HS, Panda N, et al. The Risk of Postoperative Complications After Major Elective Surgery in Active or Resolved COVID-19 in the United States. Ann Surg. 2022;275(2):242-6.

17. Jonker PKC, van der Plas WY, Steinkamp PJ, Poelstra R, Emous M, van der Meij W, et al. Perioperative SARS-CoV-2 infections increase mortality, pulmonary complications, and thromboembolic events: A Dutch, multicenter, matched-cohort clinical study. Surgery. 2021;169(2):264-74.

18. Cardiothoracic Interdisciplinary Research Network, COVIDSurg Collaborative. Early outcomes and complications following cardiac surgery in patients testing positive for coronavirus disease 2019: An international cohort study. J Thorac Cardiovasc Surg. 2021;162(2):e355-e72.
